# Effects of Age and Muscle Activation on Three-Dimensional Spine Kinematics and Asymmetry in Elderly Adults

**DOI:** 10.3390/jcm14051610

**Published:** 2025-02-27

**Authors:** Asghar Rezaei, Chih-Hsiu Cheng, Robert J. Pignolo, Lichun Lu, Kenton Kaufman

**Affiliations:** 1 Department of Orthopedic Surgery, Division of Orthopedic Research, Mayo Clinic, Rochester, MN 55905, USA; rezaei.asghar@mayo.edu (A.R.); lu.lichun@mayo.edu (L.L.); 2 Department of Physiology and Biomedical Engineering, Mayo Clinic, Rochester, MN 55905, USA; 3 School of Physical Therapy and Graduate Institute of Rehabilitation Science, College of Medicine, Chang Gung University, Taoyuan 33302, Taiwan; chcheng@mail.cgu.edu.tw; 4 Bone and Joint Research Center, Chang Gung Memorial Hospital, Taoyuan 33333, Taiwan; 5 Department of Medicine, Divisions of Geriatric Medicine and Gerontology, Endocrinology, and Hospital Internal Medicine, the Robert and Arlene Kogod Center on Aging, Mayo Clinic, Rochester, MN 55905, USA; pignolo.robert@mayo.edu

**Keywords:** range of motion, kinematic asymmetry, EMG, trunk motion, aging

## Abstract

**Background/Objectives:** Limited spinal range of motion (ROM) is linked to low back disorders, emphasizing the need to maintain mobility in the elderly. This study measured maximum spinal ROM, asymmetrical patterns, and the effects of age and muscle activation on spinal mobility. **Methods:** Forty healthy participants aged 50 and older were recruited. An optical motion capture system recorded three-dimensional coordinates of reflective markers placed on spinal landmarks. Asymmetry was analyzed in sidebending and axial rotation. Electromyography (EMG) data were collected bilaterally from paraspinal muscles at L3 during flexion, extension, sidebending, and axial rotation. **Results:** Trunk ROM averaged 111° in flexion, 38° in extension, 46° in sidebending, and 87° in axial rotation. Kinematic asymmetry was observed in sidebending and axial rotation. ROM decreased with age in flexion motion (*p* ≤ 0.04). EMG activity was significantly correlated with ROM data for all combined motions (*p* = 0.0002). The strongest EMG signal was recorded during flexion, whereas the weakest signal was observed during extension. EMG activity also correlated with kinematic asymmetry (*p* ≤ 0.03). **Conclusions:** Age and muscle activation significantly influence spinal ROM in the elderly. Lumbar kinematic asymmetry can be partially attributed to paraspinal muscle activation, underscoring the importance of analyzing asymmetrical motions in conjunction with EMG activity.

## 1. Introduction

Physical activity declines with age, degenerative pathologies become more common, and, as a result, the maximum spinal range of motion (ROM) is reduced [1,2]. In clinical practice, the assessment of spinal motion and lordosis are two common examinations performed to evaluate patients with low back pain (LBP). Several studies have investigated the effect of age on spinal flexibility in different patient populations [3]. Previous studies used ROM analysis during physical activities to identify patients with LBP, even though no clear-cut measures have been proposed [4].

Clinical assessments of spinal motion usually include a variety of singular-plane measurement systems from simple goniometry to planar radiographs that expose patients to ionizing radiation [5,6,7]. However, the spinal column has a complex structure that moves in three-dimensional space during physical activity. Therefore, measuring planar motions is not a true representation of spinal motion. Additionally, these measurement techniques cannot assess patients’ mobility during activities of daily life. Measuring three-dimensional spine kinematics using a skin-based reflective marker system is an objective technique that measures spinal motions in the free-living environment with high accuracy [8,9,10,11]. Additionally, paraspinal muscle activation may have a complex impact on trunk range of motion, depending on the type of spinal movement. These muscles play an important role in the stability of the lumbar and thoracic spine and are, therefore, crucial in diagnosing spinal disorders [12].

While multiple studies have investigated age-related changes in trunk motion, the extent to which aging affects the maximum voluntary ROM of the lumbar and thoracic regions, along with their associations with muscle activation in the elderly has not been described. This study aimed to assess the maximum voluntary three-dimensional spinal motion using optical motion capture, alongside paraspinal muscle EMG signals. The study tested two hypotheses: (1) that age significantly impacts spinal mobility in the sagittal, coronal, and axial planes and (2) that age-related kinematic asymmetry exists.

## 2. Materials and Methods

### 2.1. Participants

In this cross-sectional study, healthy, asymptomatic individuals over the age of 50 were enrolled after obtaining approval from the Mayo Clinic institutional review board (approval No. 20-013160; March 2021). Participants were recruited to ensure that the number of subjects aged 50 to 64 was equal to the number of those aged 65 and older for each sex. Some individuals were recruited randomly through a flyer campaign posted on the Mayo Clinic website. Because recruiting subjects aged 65 and older proved challenging, additional participants from this age group were sourced from the Rochester-area Older Adult Registry (ROAR), a Mayo Clinic community-based, longitudinal primary care population of adults aged 65 and older. Patients signed an informed consent form before being enrolled in the study. Exclusion criteria included individuals with a BMI greater than 35, difficulty performing common activities of daily living, neuromuscular disorders, use of assistive walking devices, or frailty. Subjects with back pain, spinal pathologies, or a history of spinal surgeries or procedures were excluded based on self-reported pain and/or a review of their medical records.

Sample size: The sample size was calculated using BlueSky v10.3.1-Pro, based on normative spinal ROM data from a previous study [13]. This study employed linear regression analysis, similar to the majority of data analyses in the current paper and reported correlation coefficients of r = −0.53 for female subjects and r = −0.56 for male subjects. To achieve a statistical power of 0.8, a significance level of 0.05, and detect a Pearson correlation of r = 0.53, a minimum of 25 subjects is required to reach statistical significance.

### 2.2. Measurement of Lordosis Curvature

Lumbar lordosis curvature was measured using a 12-inch contour gauge, which was gently pressed against the lumbar spine while the subject was standing. The curvature was then transferred to a piece of paper, and the locations of the S2 and T12 were marked to measure the curvature as described previously [14].

### 2.3. Marker Placement

**Figure 1:**
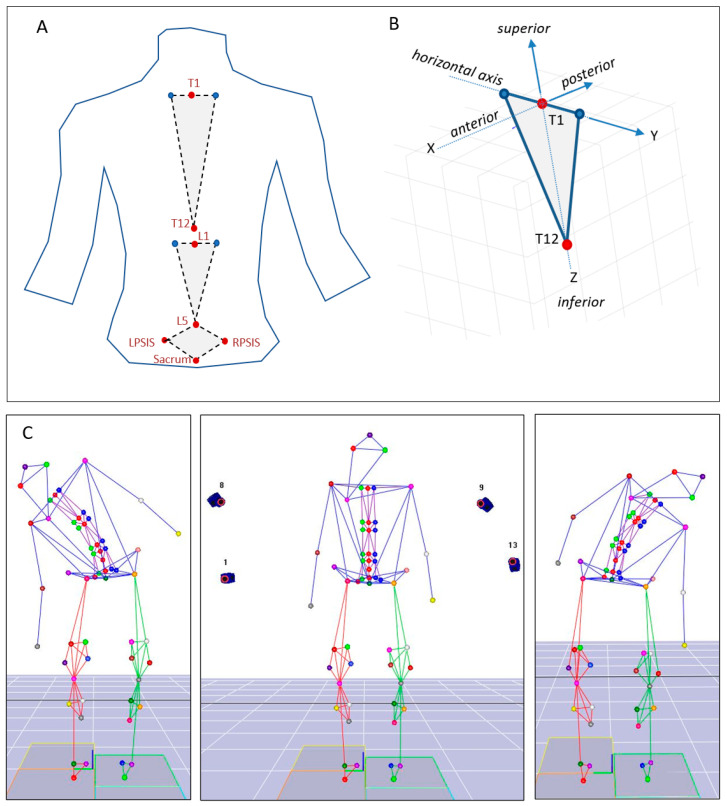
(**A**) The spine marker set including eleven reflective markers to capture kinematic motions. (**B**) Coordinate system definition for the thoracic segment and direction of axes. (**C**) Illustration of a participant represented as a stick figure with markers placed at key anatomical landmarks. Four out of fourteen motion-capture cameras are shown positioned behind the subject, recording the motion of each marker. Panels on the right and left depict sidebending activities captured during the experiment.

A total of 58 spherical reflective skin markers were attached to anatomical landmarks to define all body segments, including 11 markers specifically used to represent thoracic and lumbar spinal segments for kinematic modeling; five markers were attached over the spinous processes of T1, T12, L1, and L5, as well as on the sacrum at S2; two markers were attached over the left and right posterior superior iliac spine (PSIS) (Figure 1). Four bilateral markers were attached at T1 and L1 levels, with about 2.5 cm distance from the central marker. All marker placements were performed by trained physical therapists to ensure consistency and accuracy.

### 2.4. Kinematic Model

The trunk was divided into three segments: thoracic, lumbar, and sacrum (Figure 1A). The thoracic segment was defined with a vertical axis, ZT, from T12 to T1, a horizontal axis, YT, passing through the bilateral markers at the T1 level (Figure 1B). Similarly, the lumbar segment was defined with a vertical axis, ZL, from L5 to L1, and a horizontal axis, YL, passing through the bilateral markers at the L1 level. Finally, the sacrum segment was defined with a vertical axis, ZS, from sacrum to L5, and a horizontal axis, YS, passing through the PSIS markers. In all the segments, an anterior–posterior axis, X, was defined to be mutually perpendicular to their corresponding Y and Z axes. All mathematical modeling and data processing were performed using custom-made code in MATLAB R2020b (MathWorks, Natick, MA, USA).

Rotation around X axis was defined as lateral bending; rotation around Y axis was defined as flexion/extension, and rotation about Z axis was defined as axial rotation (Figure 1B). A widely used Cardan/Byrant sequence of flexion/extension, adduction/abduction, and axial rotation was used to calculate the angles of interest [15].

Spinal mobility was assessed as ROM of the thoracic (T1–T12 segment) relative to the lumbar (L1–L5 segment) as well as ROM of the lumbar segment relative to the sacrum. Furthermore, ROM measurements of the sacrum segment were calculated relative to the global (lab) coordinate system. The trunk ROM was defined as the cumulative sum of the ROMs from the thoracic, lumbar, and sacrum segments. Marker trajectory data were collected at 120 Hz using a 14-camera Real-Time Motion Analysis system (Raptor 12HS, Motion Analysis, Rohnert Park, CA, USA) and filtered using a second-order low-pass Butterworth digital filter [16] with a cutoff frequency of 16 Hz.

Dominant and non-dominant sidebending and axial rotation motions were compared to evaluate kinematic asymmetry during each movement. Kinematic asymmetry was calculated as the difference between dominant and non-dominant motions: *ROM_(Dominant)_* − *ROM_(Non-dominant)_*. Positive values indicate greater motion on the dominant side, while negative values indicate greater motion on the non-dominant side. The asymmetry values were then compared with the mean ROM values for the corresponding motions.

### 2.5. Measurement of Muscle Activation

Electromyography (EMG) data were collected from the paraspinal muscles bilaterally at the L3 level using surface electrodes, as previously explained in detail [17]. After cleansing the skin with alcohol swabs, two electrodes were placed approximately one inch lateral to the spine midline at the L3 level [18], and a reference electrode was positioned on the lower left back. Muscle quality at the L3 level is commonly used as a key indicator to assess overall skeletal tissue health and to examine the effects of reduced muscle mass on various chronic diseases [19,20].

Muscle activation signals were recorded at 2400 Hz and digitally band-pass filtered between 20 Hz and 450 Hz using a fourth-order Butterworth filter. The signals were then full-wave rectified, and the root mean squared (RMS) value was calculated to quantify muscle activation. The muscle activation threshold was set at 2.5 standard deviations above the quiescent muscle activity level during a quiet standing task [21]. EMG data were then normalized to this threshold to obtain amplitude-normalized muscle activation, which was subsequently divided by the task duration to calculate time-normalized muscle activation. Finally, the normalized muscle activation values were averaged over three repeated trials for analysis.

### 2.6. Experimental Protocol

The dominant side was determined based on the preferred leg for kicking a ball and the preferred hand for writing. The subjects were instructed to stand still and relaxed with eyes looking straight forward for thirty seconds to establish a quiescence level. Next, six spinal movements were performed to assess ROM data. These tasks included forward flexion and extension in the sagittal plane, sidebending to the dominant and non-dominant sides in the coronal plane, and axial rotation to the dominant and non-dominant sides in the transverse plane. Each task was repeated five times, and the three middle trials were used for analyses.

### 2.7. Statistical Analysis

BlueSky v10.3.1-Pro was used for all the statistical analysis. Simple regression analysis was employed to determine the significance of age and EMG on spinal ROM. T-tests were used to compare BMI values between both sexes. During sagittal and transverse mobility, EMG signals from both the dominant and non-dominant sides were averaged, and these average values were used for analysis. For coronal mobility, only the EMG signals from the side corresponding to the direction of motion were included in the regression analysis, reflecting differences in muscle function during sidebending. A linear mixed model was used to evaluate the relationship between EMG and ROM data across all combined motions, with EMG, motion type, and their interaction as explanatory variables, and ROM as the outcome. Average values and standard deviations were reported. Statistical significance was set at 0.05.

## 3. Results

Forty eligible participants aged between 51 and 82 years (mean age: 65 ± 10 years) were recruited (Table 1). The age and BMI differences between female and male subjects were not statistically significant (*p* ≥ 0.24). Out of forty individuals, three (two females and one male) were left-dominant (left-handed and left-footed), while the remaining individuals exhibited right dominance.

### 3.1. Lordosis Curvature

The lordotic curvature was greater in the female subjects (43° *±* 19°) compared to the male subjects (39° *±* 18°), but this difference was not statistically significant (*p =* 0.42). Neither age nor sex nor BMI was able to explain the sagittal curvature variability (*p ≥* 0.15).

### 3.2. Kinematic Motions

Sagittal mobility: During flexion, the trunk ROM was 111°, lumbar ROM was 15°, and thoracic ROM was 33°. Sacrum motion was 63° during flexion (Table 2). During extension, the trunk ROM was 38°, lumbar ROM was 5°, and thoracic ROM was 17°. 

**Figure 2:**
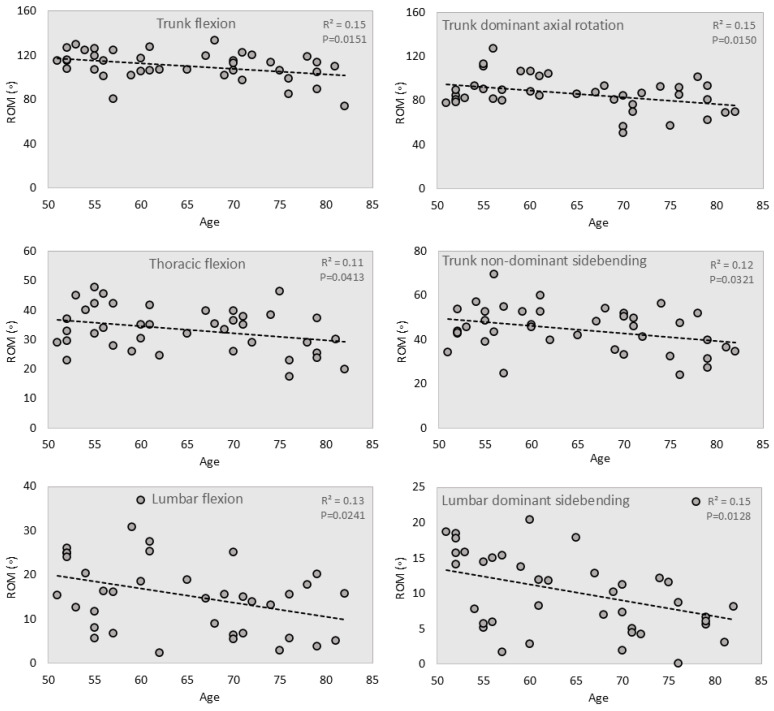
Scatterplots of ROM and age, including linear regression lines, showing that age significantly affects ROM during flexion, sidebending, and axial rotation.

Regression analyses showed that age had a significant effect on lumbar, thoracic, and trunk ROM (*p ≤* 0.0413) (Figure 2 and Table 3). Flexion ROM deteriorated by 3.2° in the lumbar region, 2.5° in the thoracic region, and 5.0° per decade in the trunk region. Sacrum flexion ROM did not vary significantly with age (*p =* 0.79). Extension ROM also did not significantly change with age (*p ≥* 0.08) (Table 3).

Coronal mobility: Coronal ROMs averaged about no more than 11° in the lumbar region, and up to 28° in the thoracic region (Table 2). The trunk ROM was up to 47° during sidebending. For the lumbar sidebending, age had a significant effect of ROM on the dominant side (*p =* 0.0321), deteriorating by 2.2° per decade (Table 3). Age also significantly affected trunk non-dominant sidebending movements, showing a decline of 3.4° per decade (*p ≤* 0.0410) (Figure 2).

Transverse mobility: The lumbar segment had a restricted motion of only 4°, while the thoracic segment motions were up to 31° (Table 2). The sacrum and the trunk rotated 54° and 86°, respectively, in the transverse plane. Except for trunk rotation on the dominant side (*p* = 0.0150), age did not significantly affect the ROM in axial rotation (*p* ≥ 0.12) (Figure 2).

**Figure 3:**
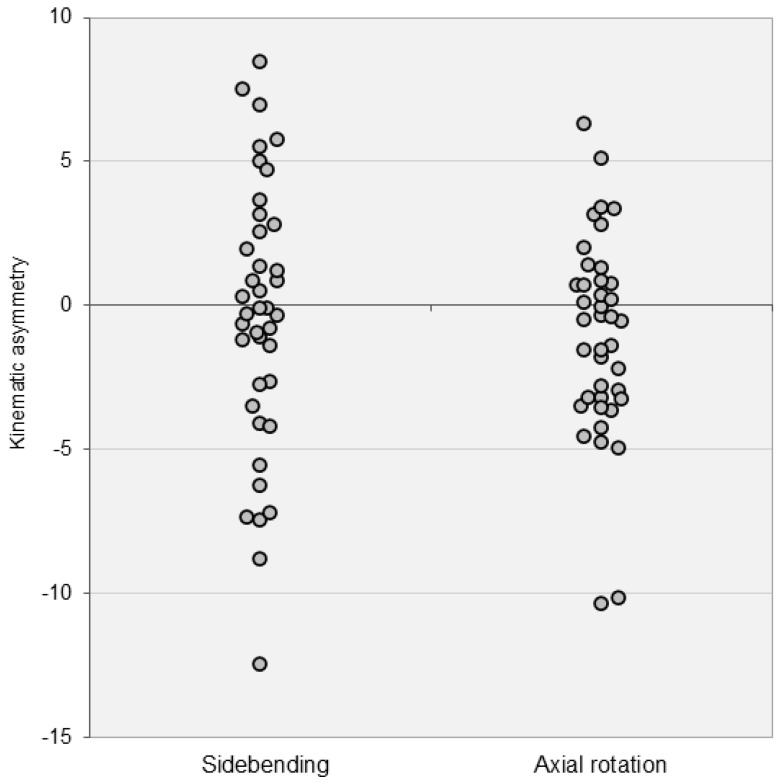
Kinematic asymmetry in sidebending and axial rotation motions. The Y-axes display the asymmetry in lumbar movements, calculated as the difference between the motion on the dominant and non-dominant sides. Positive values indicate greater movement on the dominant side, while negative values indicate greater movement on the non-dominant side.

Kinematic symmetry: Asymmetrical movements occurred during sidebending and axial rotation (Figure 3). The sidebending motion exhibited greater asymmetry compared to axial rotation. An asymmetry of 5° in sidebending represents approximately 50% of the cohort’s average ROM, which is 11° (Table 2). In axial rotation, 2° of asymmetry represents approximately 50% of the cohort’s average ROM. Using this threshold, 22 out of 40 individuals were identified as having kinematic asymmetry in the transverse plane.

### 3.3. Muscle Activation

Muscle activations from both sides were recorded during each task. On average, flexion motion produced maximum muscle activation approximately 11 times the quiescent standing EMG activity, while extension exhibited a minimum activation of 3.2 times. Sidebending generated an EMG value of 4.3 times, and axial rotation produced a value of approximately 4.2 times the quiescent standing activity (Table 4).

**Figure 4:**
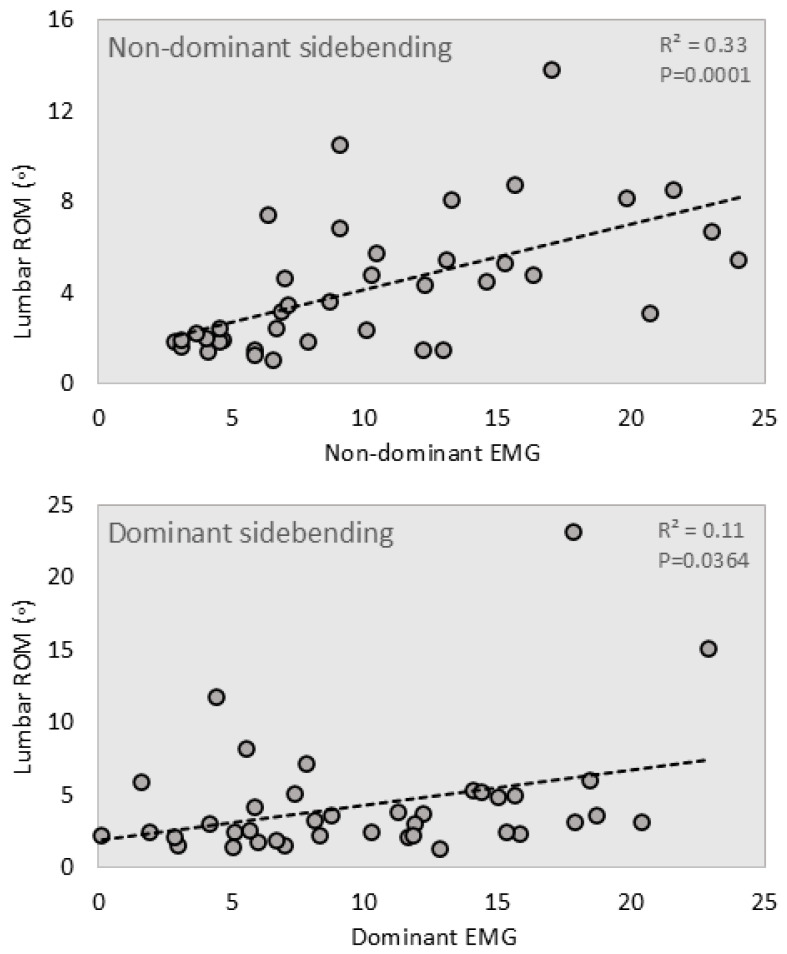
Linear regression analysis of sidebending ROM data and EMG signals, including their linear regression lines.

Regression analysis was performed using EMG signals as the explanatory variable (in place of age), with lumbar ROM as the outcome. Significant correlations were found between sidebending on the non-dominant side and paraspinal muscle activation (*p <* 0.001) (Table 4) (Figure 4). A significant correlation was also found between sidebending on the dominant side and muscle activation on the same side (*p* = 0.0364).

**Figure 5:**
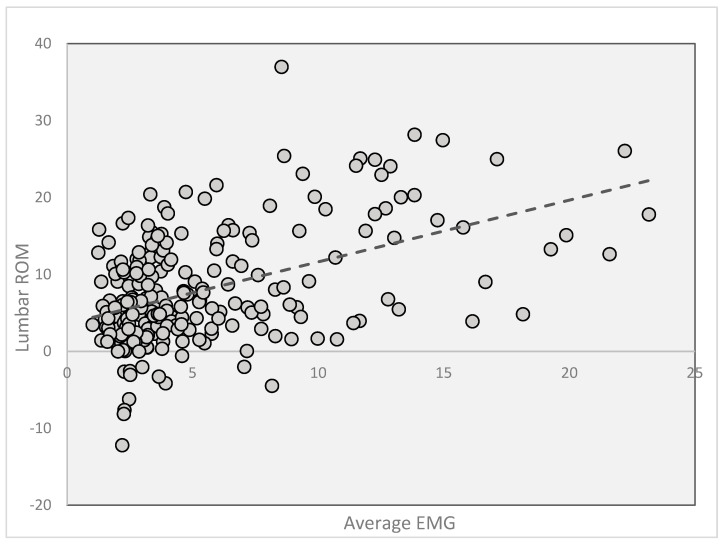
Scatterplots of average EMG and ROM data for all combined motions, accompanied by a linear regression line indicating the overall trend.

With all six spinal movements combined leading to 240 rows of data, a mixed linear model found significant effects for motion type (*p =* 0.0103) and EMG (*p =* 0.0002), but not for their interaction (*p =* 0.1). This means ROM data depend on motion type and generally increases with EMG signals (Figure 5).

**Figure 6:**
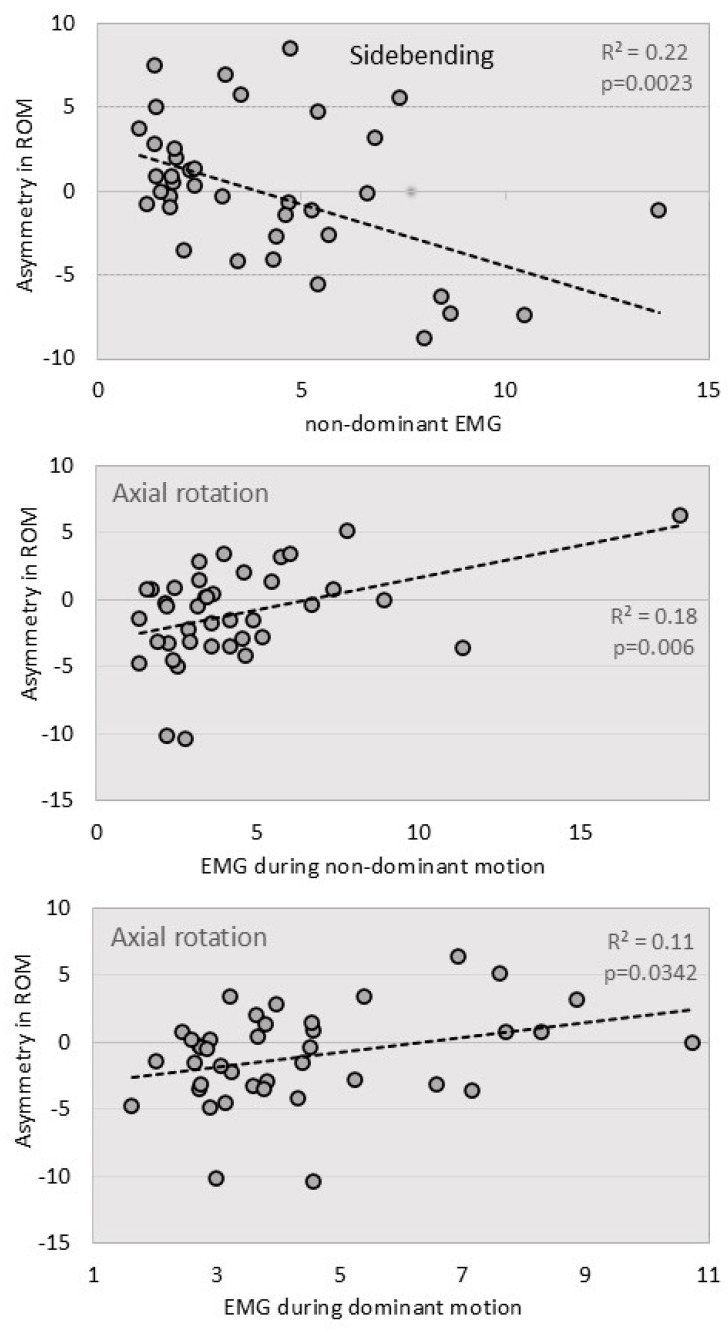
Scatterplots of kinematic asymmetry and muscle activation, with corresponding linear regression lines.

*EMG and kinematic asymmetry:* For sidebending, a significant correlation was found between kinematic asymmetry and muscle activation recorded during non-dominant motion (*p =* 0.0023), suggesting stronger EMG signals in individuals with larger ROM on their dominant side. For axial rotation, kinematic asymmetry showed significant correlations with average EMG data recorded during both non-dominant motion (*p =* 0.006) and dominant motion (*p =* 0.0342). However, unlike sidebending, EMG signals were stronger in individuals with larger ROM on their non-dominant side. Figure 6 illustrates these relationships between kinematic asymmetry and EMG signals.

## 4. Discussion

This study assessed the effect of age and muscle activation on the maximum voluntary spinal ROM during tasks of daily living performed by elderly subjects. There was a significant decline in spinal mobility during flexion, while aging had a lesser impact on other motions. Muscle activation significantly affected sidebending motion. When all motions were combined, higher muscle activation resulted in larger ROM. Extension exhibited the weakest EMG signals, approximately 3.2 times stronger than the quiescent signal, while flexion showed the strongest signals, about 11 times stronger than the quiescent signal. Kinematic asymmetry was found during both sidebending and axial rotations showing correlations with average muscle activation.

The current study revealed that lumbar ROM declined with age in both the sagittal and coronal planes. Notably, both women and men experienced an average loss in the lumbar region of more than 3° per decade during forward bending. When compared to the baseline ROM at age 50 in our cohort, healthy elderly patients may encounter a limited ROM in flexion of about 48% over three decades. This reduction in ROM, combined with decreased muscle strength [22], contributes to locomotion disability by causing movement-related functional limitations, significantly affecting independence and quality of life in older adult populations [23].

The lumbar spine experiences significant loads during physical activities while undergoing three-dimensional motions. In contrast, the thoracic spine experiences lower loads and reduced ROM due to its attachments to the rib cage. This structural difference may contribute to the higher prevalence of degenerative changes in lumbar intervertebral discs compared to thoracic ones [24]. A previous in vitro study assessed lumbar intervertebral disc ROM and found that age-related disc degeneration led to decreased disc height and potentially reduced ROM [25]. Subjects with severe lumbar disc degeneration exhibited limited segmental motion, comparable to the restrictions seen in spinal fusion [26]. Lumbar ROM is a critical measure in the elderly, as previous studies have shown a direct relationship between lumbar mobility and functional performance [27]. These findings align with the current study, which divided the spine into thoracic and lumbar segments, and suggest that age significantly reduces spinal ROM. These results highlight the importance of considering limited ROM alongside the degree of disc degeneration when assessing spinal health in aging populations.

In segments with reduced ROM, stiffness increases at the end range, potentially leading to structural tissue damage by resisting further movement and increasing compressive forces on the vertebrae [28]. Additionally, vertebral structural integrity decreases during sagittal and coronal mobility [29]. Therefore, reduced ROM may be a risk factor for the elderly, as age-related bone loss further compromises spinal health, exacerbating susceptibility to fractures and degenerative changes.

Multiple studies have suggested that asymmetrical movements could be indicative of LBP [30,31,32]. However, in the current study, asymmetrical patterns were observed in sidebending and axial rotations among asymptomatic subjects. About 14 individuals (35% of the cohort) exhibited noticeable side differences of at least 50% of the average ROM during sidebending. For axial rotation, 55% of individuals showed asymmetry of at least 50% of the average ROM. These asymmetries may result from habitual movement preferences favoring one side over the other. Sidebending is crucial for postural adjustment and maintaining balance during activities such as reaching sideways. Muscle activation during this motion may indicate the role of neuromuscular control in spinal mobility. Additionally, the asymmetrical patterns observed during sidebending may result in compensatory movements or uneven loading, potentially contributing to spinal instability or LBP over time.

While multiple definitions of asymmetry exist in the literature, asymmetry may vary across indices, potentially confounding outcomes [33,34]. These studies have proposed individualized asymmetry indices for more accurate interpretation. However, no universally accepted asymmetry threshold currently exists to alert patients or clinicians to spinal pathologies related to asymmetry. Consequently, we did not use any specific asymmetry index to represent spinal kinematic asymmetry in this cohort and refrained from defining a threshold. Instead, we reported differences in the ROM between dominant and non-dominant sides [35], comparing them with the cohort’s mean values to highlight clinical significance and raise awareness. This highlights the need for an individualized approach to selecting an appropriate asymmetry index for spinal kinematic asymmetry, as well as the establishment of an objective threshold to interpret such data for clinical applications.

Reduced ROM may have multiple implications, including accelerated degeneration of neighboring segments due to altered spinal loading [36]. It can also lead to muscle weakness, as muscles do not function through their full potential, which may exacerbate muscle atrophy and contribute to the development of LBP [37]. Among various exercise therapies for chronic LBP, strengthening exercises have been found to be the most effective for improving physical function [38]. Participants in our study (average age: 65), though asymptomatic, exhibited limited ROM in the lumbar and thoracic regions. With the growing aging population, facilitated by advancements in technology and healthcare, such limited ROM may predispose individuals to LBP or functional disability in later years. Screening for ROM and trunk strength, along with exercise recommendations in the healthy elderly population, may serve as effective preventative measures against LBP. Furthermore, corrective spinal motions may help mitigate kinematic asymmetry and reduce the risk of age-related spinal pathologies.

Paraspinal muscles play a crucial role in the trunk stability and movements of the lumbar spine [39]. These muscles are typically smaller in patients with LBP compared to healthy individuals [40]. The current study measured muscle activation in healthy subjects during spinal movement across different planes, demonstrating that larger ranges of motion (ROM) required stronger muscle activation, with flexion showing the highest activation. Importantly, this study revealed that muscle activation was significantly correlated with lumbar sidebending motion, and stronger signals were associated with larger ROM. More importantly, this study identified a correlation between muscle activation and asymmetrical movements. These findings suggest that asymmetrical motions should be analyzed alongside EMG data to gain a more comprehensive understanding of spinal disorders. Additionally, the correlation between muscle activation and asymmetrical movements suggests that neuromuscular imbalances may play a critical role in the development and progression of spinal disorders, including LBP, scoliosis, and degenerative spinal conditions. Monitoring these movement and muscle activation imbalances could aid in the early detection of spinal dysfunction, potentially before structural damage occurs. Rehabilitation programs tailored to correct asymmetrical muscle activation may help restore balanced movement patterns.

Optical motion capture systems are a reliable and valid technique for measuring 3D spine kinematics [41]. While numerous studies utilizing optical motion capture systems have focused on activities of daily living such as walking, running, or sit-to-stand movements [10,42], no studies have been performed that specifically measure the maximum voluntary spinal motion during tasks involving flexion/extension, sidebending, and axial rotation in the elderly and report the association with muscle activation. Consequently, we were unable to compare the maximum spinal ROM of the elderly individuals in this study with other reports in the literature using the same technique.

While previous studies have generally shown greater flexibility of the lumbar region compared with the thoracic [5], the current study on an elderly cohort revealed greater mobility in the thoracic region than in the lumbar region. These differences can partially be attributed to variations in methods and protocols employed to define and measure ROM [3]. To shed light on these discrepancies, we measured the ROM of the sacrum and trunk for all tasks. The trunk ROM during flexion in our study was 111°. The trunk ROMs in this study compared to those reported in previous studies accounted for the majority of the discrepancies observed. For instance, Narimani and Arjmand [43] used inertial tracking devices to measure spine ROMs, defining the lumbar segment as T12-S1, and reported a flexion ROM of 55° for the lumbar region. They also measured trunk ROMs of 119°. In contrast, our study, which considered L1–L5 as the lumbar segment, yielded significantly smaller lumbar ROMs of approximately 15° during flexion. This discrepancy primarily stems from our exclusion of the sacrum as part of the lumbar region. Another study conducted by Kienbacher et al. [44] measured a trunk ROM of 110° during flexion, similar to our findings, but their lumbar ROMs during flexion were significantly greater than that in the current study. This difference comes from Kienbacher et al. defining lumbar ROM as the difference in orientation between the T4 and L5 segments in maximum flexion and standing positions. Previous studies have also highlighted disparities in techniques and definitions, making the comparison of spinal curvatures and ROMs challenging [45]. These observations highlight the need for standardized ROM measurements to enable unbiased comparisons.

The skin-mounted marker technique is sufficiently accurate for capturing kinematic motion [46] and can adequately track the position and orientation of the vertebrae compared to MRI [47,48]. However, this technique has inherent limitations, such as soft tissue artifacts, which may be exacerbated by BMI. While some studies have found no clear relationship between BMI and soft tissue artifacts [47], the results from subjects with a high BMI should be interpreted with caution. Consistent with previous studies, we excluded subjects with a BMI greater than 35 kg/m^2^ [49]. Since the optical motion capture system non-invasively captures kinematic motions in free-living environments, it is essential to standardize its applications, investigate its limitations, and address the effects of soft tissue artifacts to optimize its use for targeted populations.

This study has several limitations. We employed a motion capture system to assess spinal ROM; however, this technique does not directly measure intervertebral segmental motion. Instead, it captures the global motion of spinal regions. Therefore, these results should be interpreted in the context of functional spinal movement rather than as direct segmental ROM measurements. Future studies integrating imaging-based motion analysis, such as fluoroscopy or MRI, could provide additional validation. The spine was modeled as three segments: the sacrum, lumbar and thoracic regions. Treating the entire thoracic or lumbar region as a single rigid segment may not be the most accurate assumption. Yet, it allowed us to record all the motions reliably. Further, since a cross-sectional study design was employed, there is potential for selection bias. Additionally, we did not measure passive ROM during spinal movements, which could have offered more insight into the effects of EMG signals and their relationships to kinematic ROM. Certain subgroups of the population may have been likely to participate, leading to a potentially unrepresentative sample.

## 5. Conclusions

In conclusion, this study offers valuable insights into spinal motion in the elderly, highlighting the importance of considering age and muscle activation in the assessment of spinal health. This study revealed asymmetrical patterns of spinal kinematics, emphasizing the need for further investigation into the role of muscle activation during dominant and non-dominant motions. Additionally, this study underscores the necessity of establishing a standardized protocol for objectively defining and measuring spinal motion, ensuring consistency and comparability across future studies.

## Figures and Tables

**Table 1:** Demographic data of the subjects.

	Male	Female	*p*-Value
**Participants**	20	20	-
**Age (years)**	66.9 (10.0)	63.1 (10.0)	0.24
**Height (cm)**	176.8 (6.7)	162.9 (6.4)	<0.001
**Weight (kg)**	84.6 (12.2)	71.5 (10.5)	<0.001
**BMI (kg/m^2^)**	27.0 (3.5)	27.0 (4.0)	0.96

**Table 2:** Average ROM values (SD) during different tasks for female and male subjects separately and all together.

Tasks		Sacrum	Lumbar	Thoracic	Trunk
Flexion	Female	64(10)	14(8)	35(7)	113(13)
	Male	61(15)	17(9)	31(7)	108(14)
	All	63(13)	15(9)	33(8)	111(13)
Extension	Female	16(8)	5(8)	17(8)	38(12)
	Male	16(7)	5(6)	17(8)	38(10)
	All	16(7)	5(7)	17(8)	38(11)
Non-dominant sidebending	Female	7(3)	10(5)	27(8)	44(9)
	Male	8(5)	12(7)	25(10)	45(11)
	All	8(4)	11(6)	26(9)	44(10)
Dominant sidebending	Female	7(2)	11(5)	30(8)	48(10)
	Male	10(6)	10(6)	26(6)	45(8)
	All	9(5)	10(6)	28(8)	47(9)
Non-dominant axial rotation	Female	49(13)	4(6)	28(9)	80(14)
	Male	58(12)	4(2)	32(8)	93(13)
	All	54(13)	4(4)	30(9)	87(15)
Dominant axial rotation	Female	49(13)	4(4)	29(8)	82(15)
	Male	55(11)	4(3)	32(7)	90(15)
	All	52(12)	4(4)	30(8)	86(16)

**Table 3:** Results of simple regression analyses with age as the explanatory variable and ROM during various spinal movements as the outcome measures.

Tasks	ROM	Age
Flexion	sacrum	0.79
	lumbar	0.0241 *
	thoracic	0.0413 *
	trunk	0.0151 *
Extension	sacrum	0.50
	lumbar	0.08
	thoracic	0.39
	trunk	0.19
Non-dominant Sidebending	sacrum	0.78
	lumbar	0.06
	thoracic	0.33
	trunk	0.0321 *
Dominant Sidebending	sacrum	0.46
	lumbar	0.0128 *
	thoracic	0.73
	trunk	0.15
Non-dominant axial rotation	sacrum	0.61
	lumbar	0.19
	thoracic	0.14
	trunk	0.16
Dominant axial rotation	sacrum	0.12
	lumbar	0.13
	thoracic	0.08
	trunk	0.0150 *

*Asterisks indicate statistically significant differences in age (e.g., *p < 0.05).

**Table 4:** Averages (SD) of dominant and non-dominant muscle EMG data (first column), along with the results of linear regression analysis between ROM and average EMG.

Tasks	Average EMG Signal	Average EMG (*p*-Value)
Flexion	11.2 (6.3)	0.09
Extension	3.2 (3)	0.41
Non dominant Sidebending ^†^	4.3 (3)	0.0001 *
Dominant Sidebending ^†^	4.4 (4)	0.0364 *
Non-dominant axial rotation	4.2 (3.5)	0.80
Dominant axial rotation	4.3 (2.8)	0.34

^†^ EMG data were collected from the side corresponding to the direction of motion. *Asterisks indicate statistically significant differences in age (e.g., *p < 0.05).

## Data Availability

The datasets used and/or analyzed during the current study are available from the corresponding author upon reasonable request.
